# Specific Autoantibodies in Neovascular Age-Related Macular Degeneration: Evaluation of Morphological and Functional Progression over Five Years

**DOI:** 10.3390/jpm11111207

**Published:** 2021-11-16

**Authors:** Michelle Prasuhn, Caroline Hillers, Felix Rommel, Gabriela Riemekasten, Harald Heidecke, Khaled Nassar, Mahdy Ranjbar, Salvatore Grisanti, Aysegül Tura

**Affiliations:** 1 Department of Ophthalmology, University Hospital Schleswig-Holstein, University of Lübeck, 23562 Lübeck, Germany; caroline.hillers@student.uni-luebeck.de (C.H.); felix.rommel@uksh.de (F.R.); khaled.nassar@uksh.de (K.N.); eye.research101@gmail.com (M.R.); Salvatore.Grisanti@uksh.de (S.G.); Ayseguel.Tura@uksh.de (A.T.); 2 Laboratory for Angiogenesis & Ocular Cell Transplantation, 23562 Lübeck, Germany; 3 Clinic of Rheumatology and Clinical Immunology, University Hospital Schleswig-Holstein, University of Lübeck, 23562 Lübeck, Germany; Gabriela.Riemekasten@uksh.de; 4 CellTrend GmbH, 14943 Luckenwalde, Germany; heidecke@celltrend.de

**Keywords:** autoantibodies, age-related macular degeneration, biomarkers, AT1-receptor, PAR1, VEGF-A, VEGF-B, VEGF-receptor 2

## Abstract

(1) Background: Altered levels of autoantibodies (aab) and their networks have been identified as biomarkers for various diseases. Neovascular age-related macular degeneration (nAMD) is a leading cause for central vision loss worldwide with highly variable inter- and intraindividual disease courses. Certain aab networks could help in daily routine to identify patients with a high disease activity who need to be visited and treated more regularly. (2) Methods: We analyzed levels of aab against Angiotensin II receptor type 1 (AT1-receptor), Protease-activated receptors (PAR1), vascular endothelial growth factor (VEGF) -A, VEGF-B, and VEGF-receptor 2 in sera of 164 nAMD patients. In a follow-up period of five years, we evaluated changes in functional and morphological characteristics. Using correlation analyses, multiple regression models, and receiver operator characteristics, we assessed whether the five aab have a clinical significance as biomarkers that correspond to the clinical properties. (3) Results: Neither the analyzed aab individually nor taken together as a network showed statistically significant results that would allow us to draw conclusions on the clinical five-year course in nAMD patients. (4) Conclusions: The five aab that we analyzed do not correspond to the clinical five-year course of nAMD patients. However, larger, prospective studies should reevaluate different and more aab to gain deeper insights.

## 1. Introduction

Specific autoantibodies (aab) are known to be associated with autoimmune diseases. However, several studies showed elevated levels of aab also in healthy donors who never develop inflammatory disorders [1,2]. Their exact clinical roles are still unclear but are a fascinating approach for new diagnostic and therapeutic options.

Antibody (ab) levels can be elevated or reduced in patients with inflammatory disease and healthy donors, which strengthens the idea of physiological levels and a balanced generation of aab in human physiology and pathophysiology. Riemekasten et al. introduced the term “antibodiom” to understand networks of ab levels and their interactions. Antibody levels as well as their correlations can serve as biomarkers for diseases [3]. This study is a first approach to characterize antibodies in patients with neovascular age-related macular degeneration (nAMD), in which the pathogenesis is at least partially mediated by immunological factors, including a possible autoimmune response [4].

Age-related macular degeneration (AMD) is a major cause of central vision loss worldwide and becomes even more common due to the demographic change of developed countries. Thus, along with severe individual impairment, it also causes a high economic burden to society. In nAMD, patients regularly receive intravitreal injections (IVIs) with anti-vascular endothelial growth factor therapy (anti-VEGF). Even though adverse events are rare, endophthalmitis and severe vision loss or even blindness can occur. Additionally, the regular visit rate is a high burden for elderly patients not only financially and socially but also psychologically. Besides the discomfort during the injection, especially anxiety before the treatment and fear of losing eyesight as a complication have a high impact on the patients [5]. A wide variability concerning interindividual disease progression causes several patients to be visited too frequently or not frequently enough and therefore to be over- or undertreated.

Consequently, there is a high need for markers that allow us to identify patients who need to be followed up more regularly or who can gain more individual freedom by expanding clinical visit intervals. In this study, we investigated whether certain aab are indicative of the disease current and whether they can help us differentiate between those patients.

Therefore, for this study, we analyzed aab against Angiotensin II receptor type 1 (AT1-receptor), Protease-activated receptors (PAR1), VEGF-A, VEGF-B, and VEGF-receptor 2. These factors are known to have effects on angiogenesis, which is a main pathomechanism in nAMD. AT1-receptor is a G protein-coupled receptor (GPCR) that is widely expressed in the human body. It is activated by the binding of Angiotensin II and regulates important processes, such as blood flow, sodium retention, and aldosterone secretion [6]. AT1-receptor could also promote tumor angiogenesis by inducing the VEGF expression [7]. Furthermore, AT1-receptor ab has been shown to have detrimental effects in several diseases and cause acute or chronic rejection and graft loss [8]. PAR1 is also part of the GPCR superfamily and participates in vascular development mediating the angiogenetic activity of thrombin and promoting the VEGF expression [9,10]. Vascular endothelial growth factor in turn plays a key role in nAMD pathogenesis and is the target of regular intravitreal injections. VEGF-A shows prominent activity with vascular endothelial cells, primarily through its interactions with the VEGFR1 and -R2 receptors. The latter appears to mediate almost all of the known cellular responses to VEGF. VEGF-B seems to play a role only in the maintenance of newly formed blood vessels during pathological conditions [11].

## 2. Materials and Methods

Study design and participants: Patients with active nAMD in one or both eyes were identified, and written informed consent was obtained. We excluded patients with any chronic systemic inflammatory disease. Patients with diabetic retinopathy, glaucoma, inflammatory ocular diseases, and other disorders of the vitreoretinal interface were also not considered. Blood was taken at one of the regular visits at our ophthalmological clinic. The patients were followed up monthly as part of their routine examinations following the pro re nata treatment regimen for anti-VEGF-IVIs. This study was conducted in accordance with the 1964 Declaration of Helsinki, with all participants providing written informed consent. Approval by the ethics committee of the University of Lübeck, Germany (vote reference number: 16–199) was given.

Outcome measures: The primary endpoint was the number of intravitreal injections administered over the course of one and five years. For this purpose, we reviewed the medical records of patients whose ab levels were analyzed between 2011 and 2014. We differentiated between good and poor responders; the cut-off was 6 IVIs per year or 30 IVIs over five years. Investigated secondary outcome parameters were changes in best corrected visual acuity (BCVA) and optical coherence tomography (OCT) morphology. Correct automated measurements were reviewed by at least two experienced raters and manually corrected if needed. Central retinal thickness (CRT) was automatically calculated by the OCT device using the central circle of the Early Treatment Diabetic Retinopathy Study (ETDRS) grid (Heidelberg Eye Explorer, Version 1.9.10; Heidelberg Engineering, Heidelberg, Germany). Two raters reviewed OCT images to evaluate whether intraretinal or subretinal fluid (IRF; SRF), fibrosis, or macular bleeding was present at baseline and in the five-year period.

Blood preparation: Blood was drawn into serum tubes and centrifuged at 2000× *g* for ten minutes at room temperature. The methods to measure the aab have been previously described in detail [12]. Briefly, individual serum aab were assessed using commercially available solid-phase sandwich ELISA Kits according to the manufacturer’s instructions (all CellTrend GmbH, Luckenwalde, Germany). The aab concentrations were calculated as arbitrary units (U) by extrapolation from a standard curve of five standards ranging from 2.5 to 40 U/mL. The ELISAs were validated according to the Food and Drug Administration’s Guidance for Industry: Bioanalytical Method Validation. We analyzed antibodies against AT1-receptor, Protease-activated PAR1, VEGF-A, VEGF-B, and VEGF-receptor 2.

Statistical analysis: In patients in which both eyes had active nAMD, the study eye was assigned by chance. Snellen VA was converted to logarithm of the minimum angle of resolution (logMAR) for statistical analysis. Data were analyzed using IBM SPSS (Version 24.0) and GraphPad Prism (Version 9.0). Testing for normality was done via Shapiro–Wilk test for correlation analyses and via QQ-Plots for the multiple linear regression models. Correlation analyses were carried out with Pearson’s tests and corrected for multiple testing by computing adjusted *p*-values (false-discovery rate). We carried out multiple linear and multiple logistic regressions as implemented on SPSS. Antibody levels were reflected by creating a single variable using a principal component analysis. In addition, we performed a Principal Component Analysis (PCA) to reduce the dimensionality of our independent variables. We included five independent variables (the ab mentioned above) in a correlation matrix and subsequent factor analysis (based on eigenvalues larger than one; necessary assumptions tested by KMO and Bartlett tests; Rotation: Varimax; Kaiser normalization). Our PCA resulted in two significant factor scores (based on regression analyses). Here, we used the factor score that reflected the variance observed in our data to the highest possible degree (43.3%, VEGF_R2 ab excluded). The new variable was termed “ab score”. ROC plots were plotted via SPSS, and the area under the curve (AUC) was automatically calculated. For all tests, values of *p* < 0.05 were considered statistically significant.

## 3. Results

A total of 164 eyes of 164 patients with nAMD was included. Unilateral nAMD was present in 90 patients, and the remaining 74 eyes of patients with bilateral disease were assigned by chance for further analysis. A complete five-year follow-up was achieved in 59 patients. Demographic and clinical data are summarized in Table 1. Mean age of our cohort was 78.32 years, and more women than men were included. Our patients had a mean BCVA of 0.34 logMar and CRT was 346.01 µm.

**Figure 1:**
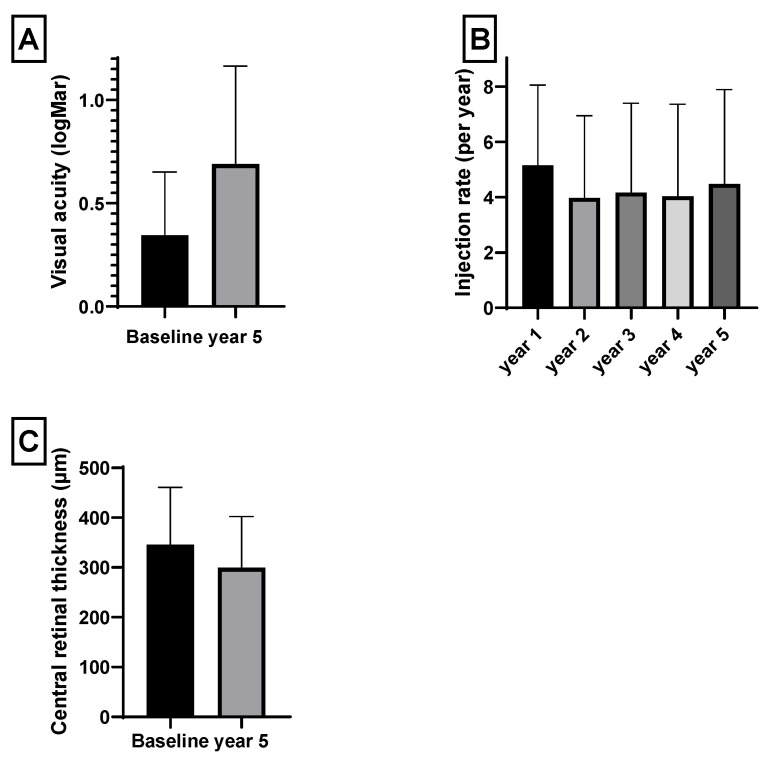
(**A**) Best corrected visual acuity (BCVA) in logMar at baseline and after five years. (**B**) Number of intravitreal injections per year. (**C**) Change in central retinal thickness.

Antibody levels were analyzed from blood samples at baseline in nAMD patients that met the inclusion and exclusion criteria and are listed in Table 2. The injection rate, BCVA, and CRT over the five years are displayed in Figure 1. Mean BCVA decreased after five years from 0.34 ± 0.31 to 0.69 ± 0.5. The mean annual injection rate remained relatively steady from year one (5.15 ± 2.91) to year five (4.48 ± 3.14). The CRT decreased from 346 ± 115 to 299 ± 103 µm. After adjusting for multiple testing, Pearson’s correlation analyses revealed no statistically significant values concerning correlation of aab with any of the clinical values. A multiple linear regression analysis was carried out to investigate whether the ab could significantly predict clinical outcome measures after five years. The results of the regression are summarized in Table 3. A multiple logistic regression analysis was carried out to analyze whether the ab predict the dichotomous clinical outcomes over five years (Table 4). Both regression models showed that neither the single aab nor taken together have a predictive value for the clinical course in the five-year time frame. We carried out ROC analyses to illustrate the diagnostic ability of the ab and ab score. The AUC values are listed in Table 5 and show low values concerning sensitivity and specificity for the evaluated aab and the clinical parameters.

## 4. Discussion

The antibodiom can serve as a biomarker for autoimmune and non-autoimmune disease, and so far, only little is known about aab in patients with nAMD. This is the first study to examine specific aab concerning their role in disease progression in nAMD patients over a five-year follow-up period.

At baseline, we included a total of 164 patients with active nAMD. Over the course of five years, we were able to analyze complete datasets of 59 eyes of 59 patients. With a mean age of 78.32 years and more women than men affected, the characteristics of our study group correspond to other epidemiological data on nAMD [13]. Intravitreal injection frequency remained steady around four IVIs per year. As we included patients with different previous disease durations, and since nAMD activity varies extremely over the years, the IVI treatment frequency in our study group is hardly comparable to other studies.

Mean BCVA at baseline was 0.34 logMar and decreased over the five-year period, as pictured in Figure 1. As patients had active nAMD with macular edema at the beginning of the study, CRT was thickened to 346.01 µm at baseline and decreased to 299.48 µm over five years. A course in BCVA and CRT like this corresponds to other data on the nAMD disease current in patients with anti-VEGF treatment [13,14,15].

As previously described, altered aab levels can occur physiologically in healthy humans who do not develop inflammatory diseases. Nonetheless, elevated or lowered aab levels can directly impact different kinds of diseases [3]. There is increasing evidence for the presence of aab in association with nAMD although it is unclear whether these ab play an active role in the etiology of the disease or if they are generated in a response to retinal injury from the underlying disease processes [16]. However, more and more evidence accentuates that autoimmunity plays an important role in the pathogenesis of AMD [16,17,18,19].

Undoubtedly, the relevance to identify specific biomarkers like aab is of high interest to the research community, as many study groups started different attempt in various study designs. Morohoshi et al. identified a pathogenic role of specific autoantibody profiles in dry and wet AMD patients [19]. They observed that ab specific to several autoantigens were significantly elevated in sera of AMD patients compared to normal controls and that these antibodies caused alterations in endothelial cell function. However, the exact clinical role and their diagnostic value towards the patient’s prognosis remains unclear. Nonetheless, this is one of the most important questions to address, as nAMD lacks specific and easily available prognostic markers. This would help us in clinical routine to identify patients who need to be followed up more regularly, as nAMD shows highly variable interindividual disease courses. The same study group around Morohoshi later focused on a microarray analysis comparing sera of AMD patients and a healthy control [20]. They detected an elevated ratio of IgG/IgM to phosphatidylserine in AMD patients that even corresponded to disease stages and also determined Vitronectin and Fibronectin as biomarker candidates. Joachim et al. and Adamus et al. also identified promising candidates in AMD patients like anti-enolase aab that even corresponded to different disease stages, or GFAP, Carboxyethylpyrrole, Cellular Retinaldehyde Binding Protein, and Retinol Binding Protein-3 [18,21]. Taking a closer look at clinical parameters and their relevance concerning the individual disease progress would be an interesting question to address in larger prospective studies. Umeda et al. conducted extensive studies on cynomolgus monkeys and identified annexin II and μ-crystallin to be elevated in early-onset macular degeneration compared to the healthy control [22].

All these studies are important steps towards larger prospective long-term studies. However, we chose five new promising candidates for aab in sera and especially focused on their diagnostic value for a long-term clinical course.

Our study examined serum levels of five different aab and reviewed medical records of the included nAMD cohort over five years. As neovascularization formation is a key characteristic of nAMD, we chose ab for our study that are known to be associated with alterations of the vascular system as described in the introduction.

We carried out a PCA to summarize these ab levels and received the ab score as described above. This model allows dimensionality reduction and enables further analyses, but it has to be noted that one ab is left out in this model, and not all ab contribute to the score to the same extent. In our multiple regression models, we were able to show that the five antibodies against AT1-receptor, PAR1, VEGF-A, VEGF-B, and VEGF-receptor 2 cannot predict clinical activity parameters for nAMD, like the number of injections, development of intra- or subretinal fluid, macular bleeding, and development of fibrosis.

We utilized ROC analyses to receive information on the diagnostic ability of the ab. Neither the single aab themselves nor the ab score were able to receive sufficient values. However, when looking at our main endpoint, the total IVI response over five years, we detected a tendency concerning AT1-receptor antibodies (AUC 0.679). Therefore, this serum marker would be particularly interesting to be investigated further in prospective studies. Pathophysiologically, the AT1-receptor, as part of the renin-angiotensin system (RAS), has been shown to play a role in angiogenesis [6]. Additionally, in mice models, ACE (angiotensin converting enzyme) inhibition with Icatibant led to significant suppression of CNV development seen in AT1-receptor knockouts [23,24]. Therefore, the RAS and its components are interesting targets for future studies, and examining further aab along with AT1-receptor ab might lead to new insights on nAMD pathophysiology.

It has to be noted that serum levels of the examined ab may be influenced by anti-VEGF intravitreal injections. Several study groups focused on systemic effects of IVI treatment and showed reduced systemic VEGF levels following an anti-VEGF injection [25,26,27,28]. Matsuyama et al. and Carneiro et al. both reported that the effect was mostly present after one day of a bevacizumab injection, but VEGF levels were still reduced even after one month [25,26]. However, in the study by Carneiro et al. and Zehetner et al., this effect was not noticeable after ranibizumab and pegaptanib injections [26,27].

Future studies should take these data into consideration and should also differentiate between the administered IVIs, as they result in varying systemic effects. In our study design, we did not differ between the different anti-VEGF agents that the patients had received in the five-year follow-up period, which may have an impact on the serum levels that we measured. However, blood was drawn on the day of an IVI before the therapy was administered. Therefore, the blood withdrawal took place at least four weeks after the last injection, which limits the systemic effect of previous anti-VEGF treatments. Additionally, it is not known to what extent the systemic VEGF levels influence the levels of our analyzed ab.

The limitations of our study include the retrospective nature, the lack of masking, and a potential selection bias, as only patients who attended monthly follow-ups for five years were included. Especially regarding the latter factor, it can be argued that patients with a high disease activity resulting in more injections and worse visual outcome tend to continue follow-ups more closely. Moreover, patients with controlled disease, good vision, and no further need for injections become lost to follow-up at our treatment center over time and continue follow-up visits at their local ophthalmologist. Additionally, as the mean age of nAMD patients is relatively high, some patients died in the five-year time frame after the study started.

In addition to these limiting factors, for this study, we only chose five aab against targets that are of high relevance in angiogenetic processes. Nevertheless, angiogenesis and neovascularization are multifactorial and complex developments that can be influenced by various pathways. Therefore, the present study only gives limited insight into the complex world of aab in nAMD and opened the door to further, more widespread study approaches.

## 5. Conclusions

We identified a relatively large group of nAMD patients that we were able to follow up over a five-year time frame after five angiogenesis-specific aab were analyzed (ab against AT1-receptor, PAR1, VEGF-A, VEGF-B, and VEGF-Receptor 2). Neither the ab by themselves nor taken together had a predictive value for the nAMD current over five years. Identifying specific ab in nAMD can help us gain a deeper understanding of the disease, which is the basis for better therapeutic strategies. The antibodiom reflects important individual processes, which makes it interesting for precision medicine concerning diagnostics as well as therapeutic approaches. Future research should focus on wider antibody networks and their levels in nAMD to gain deeper insights into this highly relevant disease entity.

## Figures and Tables

**Table 1:** Epidemiological and clinical baseline data of included patients. BCVA, best-corrected visual acuity; CRT, central retinal thickness; f, female; m, male; OD, right eye; OS, left eye; OU, both eyes; SD, standard deviation.

	*n* = 164
Age (years), mean ± SD	78.32 ± 8.17
gender (m/f)	63 (38.4%)/101 (61.6%)
Laterality (OD/OS/OU)	41 (25.0%), 49 (29.9%), 74(45.1%)
Study eye (OD/OS)	84 (51.2%)/80 (49.8%)
Baseline BCVA (logMar)	0.34 ± 0.31
Baseline CRT (µm)	346.01 ± 114.81

**Table 2:** Antibody levels in nAMD patients. ab, antibody; AT1, Angiotensin II receptor type 1; PAR1, Protease-activated receptors; VEGF, vascular endothelial growth factor.

	Antibody Level (Units/mL) *n* = 164	Standard Deviation
AT1-receptor ab	8.531	10.35
PAR1 ab	3.398	7.79
VEGF-A ab	9.262	13.96
VEGF-B ab	5.998	15.13
VEGF-receptor 2 ab	6.020	9.73

**Table 3:** Multiple linear regression model. Clinical outcomes were used as dependent variables; the analyzed antibodies served as independent variables. BCVA, best-corrected visual acuity; CRT, central retinal thickness; IVI, intravitreal injections; SD, standard deviation.

	Mean ± SD	F	R^2^	P
Number of IVIs year 1	5.15 ± 2.91	(5; 109) = 0.671	0.30	0.646
Number of IVIs year 2	3.98 ± 2.97	(5; 88) = 0.473	0.26	0.795
Number of IVIs year 3	4.16 ± 3.23	(5; 67) = 0.384	0.28	0.858
Number of IVIs year 4	4.03 ± 3.33	(5; 59) = 0.436	0.77	0.436
Number of IVIs year 5	4.48 ± 3.41	(5; 52) = 0.402	0.04	0.845
Number of IVIs total	22.42 ± 12.21	(5; 53) = 0.611	0.05	0.692
CRT change	−39.06 ± 128.686	(5; 44) = 0.686	0.72	0.636
BCVA change	0.345 ± 0.416	(5; 53) = 0.637	0.06	0.672

**Table 4:** Multiple logistic regression model. Clinical outcomes were used as dependent variables; the analyzed antibodies served as independent variables. BCVA, best-corrected visual acuity; CRT, central retinal thickness; IRF, intraretinal fluid; IVI, intravitreal injections; SD, standard deviation; SRF, subretinal fluid.

	Test Statistics	Nagelkerke’s R^2^	P
SRF development	χ^2^ (5) = 1.409	0.043	0.923
IRF development	χ^2^ (5) = 10.268	0.234	0.068
Fibrosis development	χ^2^ (5) = 4.952	0.132	0.422
Macular bleeding	χ^2^ (5) = 6.739	0.116	0.241
IVI response 1 year	χ^2^ (5) = 3.902	0.045	0.564
IVI response 5 years	χ^2^ (5) = 8.320	0.186	0.139

**Table 5:** Receiver operator characteristic. Values are given as area under the curve analysis. ab, antibody; AT1, Angiotensin II receptor 1; IRF, intraretinal fluid; IVI, intravitreal injection; PAR1, Protease-activated receptors; SRF, subretinal fluid; VEGF, vascular endothelial growth factor.

	AT1-Receptor ab	PAR1 ab	VEGF-A ab	VEGF-B ab	VEGF-Receptor 2 ab	Antibody Score
IVI response 1 year	0.443	0.522	0.416	0.418	0.435	0.430
IVI response 5 years	0.697	0.625	0.650	0.638	0.539	0.696
SRF development	0.482	0.433	0.460	0.506	0.518	0.550
IRF development	0.303	0.479	0.304	0.493	0.399	0.707
Fibrosis development	0.509	0.418	0.493	0.467	0.463	0.505
Macular bleeding	0.535	0.489	0.580	0.493	0.635	0.465

## Data Availability

Raw data were generated at the department of ophthalmology, University clinic Schleswig Holstein, Lübeck.
